# Recent Advances in Lipid-Based Drug Delivery

**DOI:** 10.3390/pharmaceutics13070926

**Published:** 2021-06-22

**Authors:** Koyo Nishida

**Affiliations:** Graduate School of Biomedical Sciences, Nagasaki University, Nagasaki 852-8501, Japan; koyo-n@nagasaki-u.ac.jp

Here, I report recent advances in lipid-based drug delivery systems, with a focus on their production, controlled drug release, targeting, and co-delivery. I also briefly discuss a novel technique for evaluating their biodistribution.

Lipid-based drug carriers are an attractive option for the delivery of various types of drugs. They include several platforms, such as liposomes, emulsions, and solid lipid nanoparticles. Exosomes, one of several extracellular vesicles, are composed of a lipid bilayer and therefore constitute a promising drug delivery tool.

Various techniques have been utilized for the production of lipid-based drug carriers. These include rehydration, ethanol injection, and reverse-phase evaporation. A novel method, namely, chloroform-injection with subsequent spontaneous-phase-transition, has been reported recently [1]. It simplifies the production of liposomes. Further, microfluidics technology combined with ethanol injection is a promising approach that improves reproducibility. The scalability of microfluidic devices can be improved by increasing the total flow rate [2]. The use of millireactors also improves scalability [3].

Successful drug delivery depends on the controlled release of the drug. To do so, various strategies have been developed that utilize stimuli-responsive and/or environment-responsive mechanisms. Liposomal bubbles with ultrasound irradiation represent a physical stimulus-responsive system that can deliver large molecules, such as plasmid DNA, to target cells [4]. In addition, increased temperature (hyperthermia) and external light can trigger local drug release. In environment-responsive systems, variations in the internal environment, such as pH, redox states, and enzymes, are used as triggers for drug release. Calcium salts, such as calcium phosphate and calcium carbonate, are pH-responsive and dissolve at an acidic pH inside endosomes. Lipid-coated calcium salt nanoparticles have also been used [5]. Further, ionizable lipids are an attractive tool for pH-responsive endosomal escape. A combination of pH- and redox-responsive mechanisms is effective at releasing mRNA into the cytosol [6]. Enzyme-triggered release of cargo is also possible.

Targeted delivery of a drug is important for efficacy and safety. Modification of lipid-based nanocarriers with ligands, such as sugars, peptides, and antibodies, is useful in target cell-selective drug delivery. External stimuli, such as magnetic fields, can also be used for targeted drug delivery [7]. Exosomes have unique characteristics that allow them to migrate to specific cells and tissues; they are therefore useful in targeted drug delivery systems [8].

Co-delivery of drugs, proteins, nucleic acids, and genes is more therapeutic due to synergistic effects [5,9]. Liposomes are a useful platform for encapsulating lipophilic and hydrophilic small molecules. Nucleic acids and genes can be complexed with liposomes via electrostatic interactions. Doxorubicin is reported to enhance the expression of co-delivered genes. Co-delivery of antioxidants with genes can also enhance gene expression.

It is necessary to develop techniques that will help elucidate the biodistribution mechanisms of lipid-based drug delivery systems, thus facilitating their advancement. A multicolor deep imaging technique that can be used to analyze the spatial distribution of lipid-based drug delivery systems in various tissues was reported recently [10]. It describes a tissue-clearing solution, namely, ‘Seebest’ (SEE Biological Events and States in Tissues), which enables rapid, effective, pH-adjustable, and lipid-preserving tissue optical clearing [10]. Such novel techniques that facilitate the development of lipid-based drug delivery systems should be developed in the future.
